# Sexual Behavior of *Drosophila suzukii*

**DOI:** 10.3390/insects6010183

**Published:** 2015-03-09

**Authors:** Santosh Revadi, Sébastien Lebreton, Peter Witzgall, Gianfranco Anfora, Teun Dekker, Paul G. Becher

**Affiliations:** 1 Chemical Ecology Unit, Department of Plant Protection Biology, Swedish University of Agricultural Sciences, Alnarp, 23053, Sweden; E-Mails: revadi.santosh@slu.se (S.R.); sebastien.lebreton@slu.se (S.L.); peter.witzgall@slu.se (P.W.); teun.dekker@slu.se (T.D.); 2 Research and Innovation Centre, Fondazione Edmund Mach, Via E. Mach 1, San Michele All’Adige (TN), 38010, Italy; E-Mail: gianfranco.anfora@fmach.it

**Keywords:** spotted wing *Drosophila*, pheromone, reproductive behavior, courtship, sexual receptivity, chemical communication

## Abstract

A high reproductive potential is one reason for the rapid spread of *Drosophila suzukii* in Europe and in the United States. In order to identify mechanisms that mediate mating and reproduction in *D. suzukii* we studied the fly’s reproductive behavior, diurnal mating activity and sexual maturation. Furthermore, we studied the change of female cuticular hydrocarbons (CHCs) with age and conducted a preliminary investigation on the role of female-derived chemical signals in male mating behavior. Sexual behavior in *D. suzukii* is characterized by distinct elements of male courtship leading to female acceptance for mating. Time of day and age modulate *D. suzukii* mating activity. As with other drosophilids, female sexual maturity is paralleled by a quantitative increase in CHCs. Neither female CHCs nor other olfactory signals were required to induce male courtship, however, presence of those signals significantly increased male sexual behavior. With this pilot study we hope to stimulate research on the reproductive biology of *D. suzukii*, which is relevant for the development of pest management tools.

## 1. Introduction

Sexual communication and behavior is fundamental for mate-finding and reproductive isolation between species [1]. Understanding sexual behavior requires knowledge of the underlying signals and how factors, such as environmental conditions, time of day, insect experience, and insect physiological, state modulate communication and behavior [2,3,4,5,6]. In particular, chemical signals and senses play important roles in insect sexual behaviors. Sex pheromones usually lead to prompt and distinct behavioral responses underlying behavioral isolation between closely related species [7,8]. Behavioral and chemical studies are of prime importance to decipher the signals, behaviors and other influencing factors that mediate reproduction in insects. Such studies have led to the discovery of many hundred insect sex pheromones and contributed significantly to our understanding of insect olfaction and odor-mediated behavior [9,10]. In *Drosophila melanogaster*, physiological, molecular and neuroanatomical data makes the olfactory circuits that detect and process the male pheromone *cis*-vaccenyl acetate one of the best investigated neuronal systems underlying odor perception [11,12,13]. Pheromone research has even been brought to practical application, and pheromone-mediated mating disruption and mass trapping are efficient and sustainable tools in integrated insect pest management [14].

The spotted wing *Drosophila*, *D. suzukii* (Matsumura) (*Diptera*: *Drosophilidae*) currently draws significant attention as an economically important fruit pest in Europe and the United States [15]. However, little attention has been paid to the sexual communication and behavior of *D. suzukii*. Studies on the sexual behavior of *D. suzukii* will contribute to our knowledge of the ecology and evolution of the fly, which may aid in the development of pest control methods.

*D. suzukii* displays characteristic courtship behavior, but to what extent the different sensory modalities are involved is not fully understood. Whereas the importance of substrate-borne vibrations generated during male courtship has been demonstrated [16], the significance of visual and pheromonal components in courtship is largely unknown. Unlike most other drosophilids, *D. suzukii* does not produce the male pheromone *cis*-vaccenyl acetate [17]. The behavioral importance of *D. suzukii* cuticular hydrocarbons (CHCs) as potential pheromones has not been shown.

Here we detail elements of mating behavior in male and female *D. suzukii*. We investigated the timing of fly mating behavior and the age at which flies become sexually active. Finally, we provide data on the production of female CHCs during the first days after emergence and, using hexane-washed CHC-free females or antennectomized males, conduct a primary assessment of their potential importance as pheromones in male mating behavior.

## 2. Experimental Section

### 2.1. Flies

The laboratory population of *D. suzukii* was established with flies collected in Northern Italy (San Michele All’Adige, Trento Province, Italy). Flies were reared on Bloomington standard cornmeal diet, at a temperature of 23 ± 2 °C and 12:12 h (light:dark) photoperiod. All flies from this rearing were of the summer morph. For testing, newly emerged flies were removed from the rearing vials; the sexes were separated under short periods of CO_2_-anesthesia and kept isolated in fresh food vials until the experiment. Experiments were performed under the same light and temperature conditions as the rearing.

### 2.2. Elements of Courtship and Mating Behavior

The reproductive behavior of *D. suzukii* was assayed using 4-d-old virgin couples. The flies were released in plastic vials (95 mm × 25 mm i.d.) or Petri dishes (55 mm i.d.) containing fly food or sponge rubber as a substrate. Fly behavior was filmed using macro lens recording (Raynox dcr-25) with a high-definition camcorder (Panasonic HDC-TM700, Hamburg, Germany). The videos were analyzed using VLC media player (open source). We recorded courtship in 82 couples; the behavior of 24 mating couples was studied in detail. We applied the catalogue of courtship elements by Spieth [18] and the framework provided by Mazzoni *et al.* [16] to describe the observed behaviors.

### 2.3. Diurnal Mating Activity

Four-day-old virgin fly couples were observed for their display of mating activity. Mating activity was quantified as the number of mating couples in four 3-h periods of the 12 h photoperiod, *i.e.*, between 6 am (lights on) and 9 am (*n* = 97 couples), 9 am–12 pm (*n* = 105 couples), 12–3 pm (*n* = 96 couples) and 3–6 pm (lights off) (*n* = 131 couples). Flies were kept in groups of 7 to 10 couples in fresh food vials. The number of copulating couples was counted for the individual 3-h periods. The number of copulating couples was initially scored per 3-h period. However, most flies appeared to initiate mating within the first 30 min of the first period (6–9 am). Therefore, we adjusted the graphing of the data collection into two sections: the first 30 min and the remaining 150 min of each period. Mating couples were removed from the vials to avoid counts of remating flies.

### 2.4. Age-Related Mating Activity and Reproduction

#### 2.4.1. Age-Related Mating Activity

Mating activity was also assayed for virgin flies of different ages, *i.e.*, 1-, 2- or 3-d-old (*n* = 6 vials containing 5 males and females of the same age). Starting with the beginning of the photoperiod (6 am), fly behavior was video-recorded for 1 h and subsequently analyzed for the number of mating couples in each replicate of the three different age groups.

#### 2.4.2. Age-Related Reproduction

Age-related reproduction was studied to determine at what age *D. suzukii* is able to produce offspring. First, we combined freshly emerged virgin males and females (*n* = 7 vials containing 5 males and females) in the morning *ca.* 2 h after the lights went on (and a sufficient number of flies had emerged), and flipped them every 12 h to fresh food vials for the following 3.5 days. Vials were kept to check for the development of larvae in relation to adult fly age.

Tests with flies of the same age reflected the age at which both sexes have reached maturity, but mating and insemination of females might take place earlier. Based on preliminary observations of mating between 1-d-old flies handled in the rearing, we assumed that insemination of females could occur within 1 day after emergence, and that males produce mature sperm within 1 day after emergence. To test these assumptions we performed additional assays of freshly emerged flies with mature 3–4-d-old flies of opposite sex. Tests started in the morning *ca.* 2 h after start of the photoperiod; freshly emerged and older mature flies were combined and kept together for 24 h.

We first combined freshly emerged virgin females with 4-d-old virgin males (*n* = 7 vials containing 5 males and females) in a similar set-up as described before. Flies were flipped every 12 h for the following 3.5 days to fresh food vials but males were removed from the vials 24 h after combining them with females. Food vials exposed to the flies were kept and examined for development of larvae.

To investigate if young males mate and are able to reproduce with mature females we slightly modified the experimental procedure and followed single couples instead of groups of flies. Freshly emerged males were combined with 3–4-d-old virgin females (*n* = 18, single couples). Males were removed at the following morning 24 h after they had been combined with the females. Females again were regularly flipped and food vials were examined for presence of larvae.

### 2.5. Courtship Assay with Hexane Washed Females

The possible impact of female CHCs on male courtship was tested using male courtship towards females that were extracted with hexane to remove CHCs on their bodies [19]. Approximately one hour prior to the courtship assay, 4-d-old females were killed by freezing. Half of the females (control) were kept in the freezer, whereas the other half was washed three times with hexane. Then, both control and washed females were placed on filter paper for thawing or evaporation of the solvent, respectively. Courtship experiments began fifteen minutes after placing the females on the filter paper and within 30 min after the start of the photoperiod.

Under the lid of a small glass Petri dish (30 mm i.d.) single control or washed flies were presented to single 4-d-old males for courting (*n* = 20 couples). Male courtship behavior (orientation with wing scissoring was counted as response) towards the dead females was observed for 20 min.

No CHCs could be detected by GC-MS (procedure described below) in an extract of 3 washed female flies.

### 2.6. Mating Assay with Antennectomized Males

Three-day-old virgin male flies were anesthetized with CO_2_ and both funiculi (with their aristae) were surgically ablated 24 h before the experiments. Males with (control) and without antennae were kept individually in food vials until the next morning, when (within 30 min after start of the photoperiod) a virgin female of same age (4-d-old) was paired with each male. Individual couples (*n* = 18) were tested for the initiation of courtship (orientation and wing scissoring), courtship latency (time until the male has shown orientation and scissoring behavior) and initiation of mating during a 60 min period. An equivalent number of antennectomized and control flies were always tested at the same time. Food vials with the individual couples were kept for *ca.* 14 d after the assay to check for offspring production as result of mating.

### 2.7. Quantitative Analysis of Female Cuticular Hydrocarbons

Virgin females were examined on the day of emergence or 1–4 days thereafter to determine the amount of hydrocarbons on their cuticle. Single females (*n* = 5 per age group) were extracted for 5 min with 100 µL hexane in presence of 200 ng heptadecenyl acetate as an internal standard. These extracts were condensed to approximately 10 µL under N_2_ and analyzed on a gas chromatograph coupled with a mass spectrometer (GC-MS; 6890 GC and 5975 MS, Agilent Technologies Inc., Santa Clara, CA, USA) equipped with a HP-5MS silica capillary column (30 m × 0.25 mm × 0.25 μm film thickness; Agilent Technologies Inc.). Helium was used as the mobile phase at an average linear flow rate of 35 cm·s^−1^. The GC oven temperature was programmed from 50 °C to 300 °C at 8 °C/min. Total CHCs were quantified by peak integration and comparison with the internal standard.

### 2.8. Statistics

We applied a Likelihood Ratio (LR) Chi-Square Test to test for effects of the time of day, male antennal ablation and female hexane washing on mating behavior. Multiple comparisons were conducted with Tukey contrasts on the number of matings during the four 3-h periods as well as for comparison of matings during the first 30 min of each period. A t-test was applied to analyze latency of courtship and duration of mating for males with or without antennae. ANOVA and Tukey’s *post-hoc* comparisons were used to study the effect of age on mating behavior, and a GLM with a gamma distribution and Tukey’s *post-hoc* test were used to compare the amount of CHCs from freshly emerged to 4-d-old females. Statistical analyses were conducted using Prism (V5.0a) or R (R 2.1.1, R Core Team, R Foundation for Statistical Computing, Vienna, Austria).

## 3. Results and Discussion

### 3.1. Description of Mating Behavior

In drosophilids, males show a species-specific courtship display before attempting to mount a female [18]. Here we describe typical elements of *D. suzukii* male courtship, as well as female behaviors observed when accepting or rejecting a mate. *D. suzukii* shows several characteristic courtship elements described for other drosophilids [18]. As for other *Drosophila* species, courtship is a dynamic process and behaviors of individual couples can vary [20]. Duration of courtship lies between few seconds and several minutes with repetitions of single behavioral elements. Therefore, the description is not a strict invariable sequence of a behavioral program. The described courtship sequence considers only linear transitions (behavior A → behavior B). Future studies might elaborate on more detailed ethograms illustrating sequences and frequencies of individual behavioral elements as done for other drosophilids [20,21].

**Figure 1:**
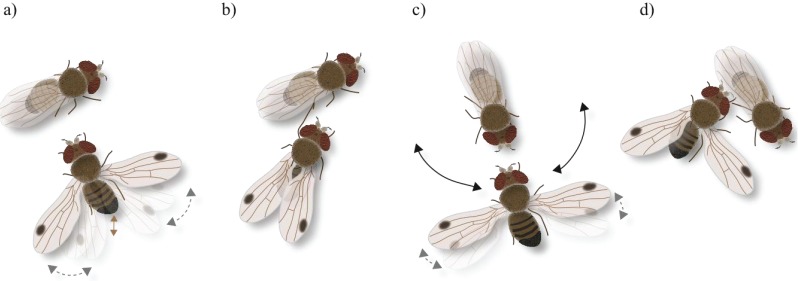
Illustration of courtship behavior in *D. suzukii*. (**a**) Male orientation, wing scissoring (grey dashed arrows) and abdominal quivering (brown arrow); (**b**) the male taps the female with his foreleg; (**c**) in front of the female the male circles to her right and left (black arrows), wings spread and sometimes flicking; and (**d**) the male then orients towards the side and rear of the female, one or both wings spread.

- Orientation: the male starts approaching the female and follows her if moving away. Fast male abdominal quivering and scissoring of the wings (see below) often accompany this initial behavior.
- Wing scissoring and flicking: when stopped, the male rapidly opens and closes both wings, sometimes with sharp flicking; wings do not fully close (semi-open) in comparison to the resting position (Figure 1a).
- Abdominal quivering: in addition to scissoring, the male rapidly vibrates the abdomen vertically, which is considered to produce distinct substrate-borne mating signals [16] (Figure 1a). During these initial behavioral elements, the female generally is not oriented towards the male (Figure 1a).
- Tapping: the male stretches a foreleg and strikes against the female abdomen, or middle or hind legs. Tapping results in either acceptance or rejection by the female, *i.e.*, heed and stay or escape, respectively (Figure 1b).
- Wing spreading and abdominal quivering: the male now is in the visual field of the female. He orients to the front of the female and when stopped, quivers with the abdomen and displays wing scissoring keeping the wings spread at approximately 180° for several seconds (Figure 1c). From time to time, sharp flicking of wings occurs. During spreading, the wings are turned to a more vertical position exposing the upper side as well as the wing spot towards the female. In between repeated spreading, the wings are not fully closed.
- Foreleg rubbing: preening of legs is observed between various courtship elements.
- Circling: positioned in front of the female the male circles, wings open and facing the female, to the female’s right and left and back to the front (Figure 1c). When the male stops at the side of the female he extends both wings, or only the one directed to the female’s front (Figure 1d). After his initial focus on the female’s front the male later circles to her rear.
- Mounting: the male bends his abdomen down and forward, comes close to the female’s abdomen and thrusts under her wings. With the forelegs, the male grasps the female’s body and mounts upon for mating.

In our recordings, the mating duration ranged from 16 to 37 min, with an average duration of 26 min.

Males were regularly courting the same female immediately after mating with her. As we observed remating in *D. suzukii* females we speculate that the meaning of postmating courtship could be to guard the female from mating with another male. Female receptivity for remating has been described in other drosophilids and is considered to affect male sexual behavior [22,23]. For males, we observed remating with another or the same female within minutes or *ca.* 3 h after the previous mating, respectively.

As in other drosophilids, males do not only court females but also males. In response, courted males generally show a wing fluttering which terminates the attempts of the courting male.

Female *D. suzukii* exhibited behavioral signals toward males, depending upon the decision to accept or reject the mate, similar to other *Drosophila* sp. [18]. Similar to males, females show preening during male courtship. The following are common behavioral responses of a female accepting the mate.

- Standstill: the female gradually stops moving and does not escape when the male is following and courting her.
- Genital spreading: the female spreads the ovipositor and moves the abdomen up and down when the male is courting.
- Ceased positioning: the female does not dip the abdomen downwards, rather holds it at resting position when the male approaches the abdomen.
- Lifting wings: the female shows a slight lifting of the wings that seems to facilitate the male’s mounting.

When mounted, the wings of the female initially are closed but then gradually spread. Towards the end of mating, we observed that females started moving, bending their abdomen and kicking the male.

We observed the following behaviors in females rejecting courting males.

- Decamping: the female flies-off, runs or jumps away from the courting male.
- Kicking: depending upon the male’s position, the female kicks the male with front-, mid- or hind-legs to avoid his close approach.
- Spin: the female vigorously spins around when the male tries to mount her.
- Abdominal depression: the female dips her abdominal tip towards the substrate.

Distinct behavioral expressions as described here generally are useful to establish behavioral paradigms for experimental assays. Courtship assays are widely used in *Drosophila* research, e.g., to study chemical signaling and chemical sensing, in intraspecific as well as interspecific context [24,25,26]. Similarly, courtship behavior could be assayed in *D. suzukii*, e.g., to understand ecological interactions with other *Drosophila* species, or to identify pheromones or other compounds that influence *D. suzukii* mating behavior.

### 3.2. Diurnal Mating Activity

*Drosophila* species differ in their genetically determined temporal activity patterns [27]. Species-specific timing of mating behavior most likely contributes to reproductive isolation between species [27,28,29]. We investigated the diurnal mating activity for *D. suzukii*.

**Figure 2:**
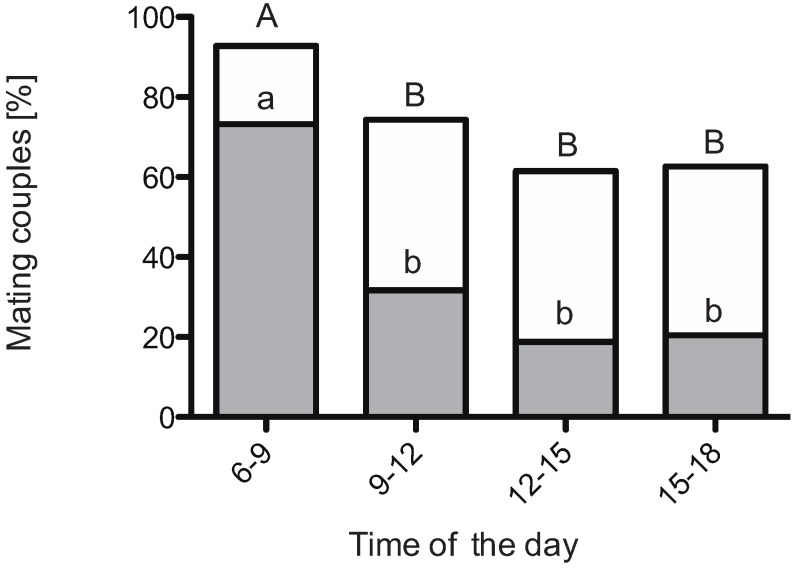
Diurnal mating activity of 4-d-old virgin *Drosophila suzukii* during four time intervals of a 12 h photoperiod. The highest percentage of mating flies was observed early in the morning, immediately after onset of the photophase. The subsections of the bars (grey) illustrates the mating events during the first 30 min of each 3 h time interval. Different letters indicate significant differences (Tukey’s multicomparison test, *p* < 0.01) within 30 min periods (lowercase letters) and within 3 h periods (capital letters).

Flies were courting and mating during all four sub-periods of the 12-h photoperiod (Figure 2). However, mating activity in *D. suzukii* varies during the course of the day. We found a strong effect of time with highest mating activity in the first 3-h period of the day (LR*χ*^2^_3_ = 37.33, *p* < 0.01; Tukey’s test, *p* < 0.01; Figure 2). For this early period we noticed that most flies had mated within the first 30 min. Data were illustrated for the first 30 min and the remaining 150 min of each period to point out the high activity in the beginning of the photoperiod (Figure 2). The significantly higher mating activity in the earliest period, consequently, was also visible when comparing the mating activity of the four 30 min intervals, (LR*χ*^2^_3_ = 75.61, *p* < 0.001; Tukey’s test, *p* < 0.001; Figure 2). Corresponding to the mating activity in the morning high locomotion activity at dawn was found for *D. suzukii* when kept under summer-day conditions [30].

### 3.3. Age-Related Mating Activity and Reproduction

The age of sexual maturation varies between *Drosophila* species [31]. Behavioral maturity and maturity of sperms or oocytes are considered as two main features of sexual maturity in *Drosophila* [31]. We studied the age-related mating activity and the age flies are able to produce offspring.

**Figure 3:**
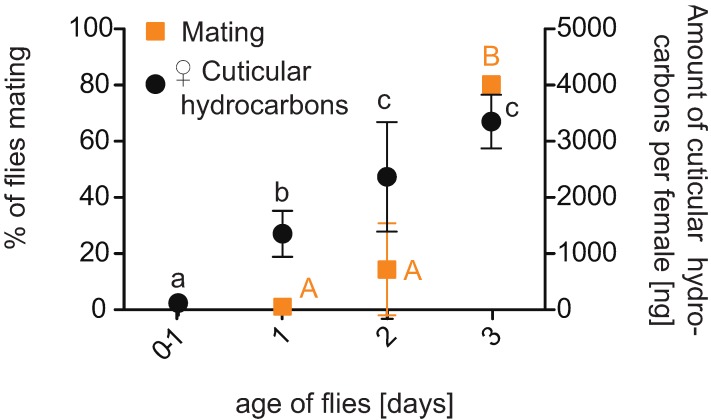
Mean percentage of mating activity of 1–3-day-old virgin flies (orange square) and development of female cuticular hydrocarbons (black circles) during 3 days after eclosion. Tested during 1 h of the main mating period (starting instantly after lights went on), virgin flies significantly increased their mating activity on the third day after emergence (different capital letters indicate significant differnce, Tukey’s test following ANOVA; *p* < 0.05, mean ± SEM). The total amount of female CHCs showed a significant increase over time (different lowercase letters indicate significant differnce Tukey’s test following GLM, *p* < 0.05, mean ± SEM).

The number of mating couples was counted during 1 h of the main mating period. No mating was observed between 1-d-old virgin males and females. However, some 2-d-old flies mated (Figure 3). At the age of three days, flies showed significantly increased mating in comparison to younger couples (ANOVA, F_2, 15_ = 31.00, *p* < 0.05; Figure 3).

In contrast to our 1-h-observations (Figure 3), we occasionally observed couples younger than 24 h mating in the fly rearing. To get a better understanding of the flies’ sexual maturation, we investigated at which age flies are able to produce offspring.

Females started producing offspring 2.5 days after emergence when kept together with males of same age (Table 1). Interestingly, an even higher percentage of females produced offspring after 2.5 days when kept with 4-d-old males for only 24 h after emergence (Table 1). The age *D. suzukii* females begin to oviposit was earlier described by a time span of one to four days after emergence [32].

**Table 1:** Age when *D. suzukii* females started producing offspring. Freshly emerged females were either continously kept with males of same age or they were kept for only 24 h with 4-d-old male *D. suzukii* (showing that females can get inseminated within 24 h after emergence by mature males)*.*

Age [Days Post Emergence] When Females Were Checked for Off–Spring Production	Percentage of Replicates (*n* = 7) in Which Females Produce Offspring	
	Freshly Emerged Females × Freshly Emerged Males	Freshly Emerged Females × 4-d-Old Males
0–2.0	0	0
2.5	43	100
3.0	57	
3.5	100	

When males were tested for sexual maturity on the day of emergence, only one out of 18 males produced offspring with a mature female.

Ovarian maturity is generally considered to correlate with the receptivity to insemination in various *Drosophila* species [33]. We showed that *D. suzukii* females could be inseminated within 24 h after emergence and thus one to two days before oviposition of fertilized eggs took place. Comparably, Kambysellis and Craddock [33] reported for two Hawaiian species insemination in previtellogenesis, close to or immediately after eclosion. Markow [34] observed mating of newly emerged female *D. melanogaster* and *D. simulans* in the laboratory and field, and suggested mating can be forced by the male.

### 3.4. Female-Derived Compounds as Possible Signals

*Drosophila* species typically differ in their social signals. Cuticular hyrdrocarbons have an especially important function in species recognition and sexual behavior [35,36,37], Particularly, female CHCs act as pheromones that mediate or enhance male courtship [38,39,40,41,42]. In addition, CHCs reflect sexual maturity of flies [36,43]. Similar to other drosophilids [36], females of *D. suzukii* increase the total quantity of CHCs during the first days after emergence (Figure 3). A significant increase of hydrocarbons on the cuticle of females was measured two to three days after emergence (GLM, F_4, 20_ = 80.84, *p* < 0.05). No further increase was measured at day 4 (3645 ± 274 ng per female; omitted in Figure 3).

The age when females had obtained their maximum measured CHCs corresponds to the age they were sexually mature and able to lay fertilized eggs (Table 1). The increase in mating activity with age was paralleled by a strong increase of hydrocarbons on the cuticle of females (Figure 3) two to three days after emergence.

We tested for possible effects of female CHCs on male behavior in a courtship assay with dead, hexane-washed females. The washing procedure to remove CHCs from the females significantly reduced the percentage of courting (orientation with scissoring) males from 70% in controls to 20% in tests with washed females (LR*χ*^2^_1_ = 10.6, *p* < 0.01). In contrast to a setting with live females, males usually did not repeat mating attempts after one or a few tries. Mating with dead females did not occur. Intensive courtship (male circling) was observed in a few males after physical contact with the dead female in controls only, indicating possible behavioral activation by CHCs via contact chemosensation [42].

**Figure 4:**
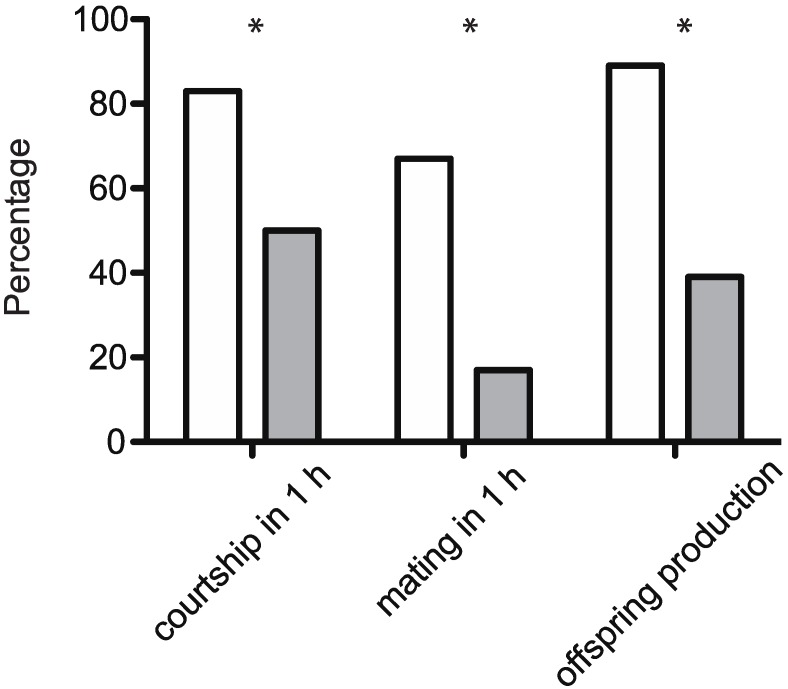
Courtship, mating and reproductive output of *D. suzukii* males with (white bars) or without (grey bars) antennae. Individual 4-d-old male-female couples (*n* = 18) were observed for one hour in the morning (starting within 30 min after lights went on). Afterwards the couples were kept together for two weeks and production of offspring was recorded. The asterisks indicate a significant decrease in courtship and mating rates as well as reduced reproductive output after antennal ablation (LR*χ*^2^, *p* < 0.01).

In *D. melanogaster*, olfactory input is important for male mating behavior [44] and short chain volatile CHCs [19] are partly detected via the antennae [45]. We therefore assayed male courtship and mating after ablation of their antennae to investigate a possible effect of female-derived olfactory signals. Antennal ablation in male flies significantly reduced the initiation of courtship behavior (LR*χ*^2^_1_ = 4.66, *p* < 0.01), mating (LR*χ*^2^_1_ = 9.77, *p* < 0.01) and the production of offspring in their female mates (LR*χ*^2^_1_ = 10.48, *p* < 0.01) (Figure 4). Ablation of antennae did not restrain all males from courtship and mating. Courtship latency was similar (t_22_ = 0.53, *p* = 0.61) for males with (20.1 ± 18.2 min) and without antennae (24.1 ± 18.4 min). Also the mating duration did not differ (t_12_ = 1.95, *p* = 0.08) for antennal-ablated males (24.0 ± 3.6 min) and control males (20.9 ± 2.1 min).

Male mating activity observed towards 3- or 4-d-old females that had built-up their CHCs was suppressed with hexane-washed females, and with antennectomized males. This indicates that female-derived compounds might be involved in sexual communication to mediate sexual behavior in males. Differences between male and female CHCs are not pronounced [17]. Significant quantitative differences between sexes were only found for few of the identified *D. suzukii* CHCs; it is therefore unclear if CHCs are involved in intraspecific mate recognition [17]. Additional experiments, such as testing if male courtship towards hexane-washed flies could be rescued by perfuming them with CHCs, are necessary to verify the behavioral activity of the potential sex pheromones and to clarify their role in mate recognition and stimulation.

Despite indications of chemosensory-mediated increase in courtship and mating, we found that males, respectively, courted and mated, to some extent, 4-d-old CHCs-free females and unmature females lacking significant amounts of CHCs. Likewise, courtship and mating was not totally abolished by males lacking antennae. Overall this suggests that neither female-derived signals detected by male antennae nor female CHCs are required to induce courtship and mating.

### 3.5. Impact on Pest Management

Understanding the mechanisms underlying sexual behavior in *D. suzukii* will improve our capacities to manage the fly as a pest.

Knowledge about temporal activity patterns can e.g., improve timing of insecticide sprays and thus efficacy [30]. The main mating time might be the best period to target both sexes at once.

The time for sexual maturation, as well as the occurrence of remating have a significant impact on the operational sex ratio [22]. Parameters of sexual maturation influence population dynamics and could contribute improving population models to guide pest management strategies [46,47]. Future work might determine sexual maturation dependent on environmental factors like temperature or host.

Knowledge of the mechanisms regulating meeting and mating of *D. suzukii* is a condition for manipulating the fly’s sexual behavior. Mating disruption is a successfully applied pest management technique against lepidopterans which however is based on long-range pheromone communication different to the sexual communication known in drosophilids.

With this current study we intend to stimulate further research on the hitherto undescribed components of sexual behavior in *D. suzukii*.

## 4. Conclusions

This study advances our knowledge on the sexual behavior of *D. suzukii*. A better understanding of the age of sexual maturation, the diurnal timing of reproductive behavior and the characterization of behavioral elements provide a foundation to study the mechanisms underlying sexual communication and behavior of *D. suzukii* in more detail. Understanding the role of chemical signals in mate recognition and sexual stimulation requires additional investigations. Further studies on the reproductive biology are relevant for control of *D. suzukii* as a pest insect.
